# Sliding Motility, Biofilm Formation, and Glycopeptidolipid Production in *Mycobacterium colombiense* Strains

**DOI:** 10.1155/2015/419549

**Published:** 2015-05-28

**Authors:** Milena Maya-Hoyos, John Leguizamón, Leonardo Mariño-Ramírez, Carlos Y. Soto

**Affiliations:** ^1^Chemistry Department, Faculty of Sciences, Universidad Nacional de Colombia, Carrera 30, No. 45-03, Ciudad Universitaria, Bogotá, Colombia; ^2^Computational Biology Branch, NCBI, NLM, NIH, Bethesda, MD 20894-6075, USA; ^3^Pan American Bioinformatics Institute, Santa Marta, Magdalena, Colombia

## Abstract

*Mycobacterium colombiense* is a novel member of the *Mycobacterium avium* complex, which produces respiratory and disseminated infections in immunosuppressed patients. Currently, the morphological and genetic bases underlying the phenotypic features of *M. colombiense* strains remain unknown. In the present study, we demonstrated that *M. colombiense* strains displaying smooth morphology show increased biofilm formation on hydrophobic surfaces and sliding on motility plates. Thin-layer chromatography experiments showed that *M. colombiense* strains displaying smooth colonies produce large amounts of glycolipids with a chromatographic behaviour similar to that of the glycopeptidolipids (GPLs) of *M. avium*. Conversely, we observed a natural rough variant of *M. colombiense* (57B strain) lacking pigmentation and exhibiting impaired sliding, biofilm formation, and GPL production. Bioinformatics analyses revealed a gene cluster that is likely involved in GPL biosynthesis in *M. colombiense* CECT 3035. RT-qPCR experiments showed that motile culture conditions activate the transcription of genes possibly involved in key enzymatic activities of GPL biosynthesis.

## 1. Introduction

The* Mycobacterium avium* complex (MAC) is widely distributed in soil and water [1], and this complex has been frequently identified as an infectious agent in animals and humans [2, 3]. The MAC comprises the species* M. avium*,* M. intracellulare* [3],* M. colombiense* [4],* M. chimaera* [5],* M. marseillense*,* M. timonense*,* M. boucherdurhonense *[6],* M. vulneris *[7]*, M. arosiense *[8], 4 subspecies of* M. avium *[9], and “MAC-other” species [10]. The opportunistic infections generated from mycobacteria in HIV-infected and immunosuppressed patients have recently highlighted the clinical relevance of MAC [11, 12].* M. colombiense* was originally isolated from HIV-positive patients in Bogotá, Colombia [4]. This species is responsible for lymphadenopathy in immunocompetent children in Spain and France [13, 14] and has recently been associated with pulmonary infections that complicate cases of cystic fibrosis [15] and disseminated coinfections with cytomegalovirus [16].

Urease-positive tests and the mycolic acid pattern by thin-layer chromatography (TLC) demonstrate the phenotypic characteristics that distinguish* M. colombiense* from other MAC species [4]. We recently used TLC to show that the mycolate profile of* M. colombiense* is characterised by the presence of mycolates I (*α*-mycolate), IV (ketomycolate), and VI (carboxymycolate) and two additional spots with chromatographic behaviours similar to those of mycolates III (hydroxyl-mycolate) and VI [17]. A unique* 16S* rDNA and the internal transcribed spacer (ITS), MAC-X, facilitated the classification of* M. colombiense* as a novel sequevar [4]. We also identified a 450-bp exclusive genomic region suitable for* M. colombiense* identification through PCR [17].

The physiological and molecular bases for MAC virulence have not been entirely established. However, the virulence of MAC strains has been associated with variations in colony morphology [18, 19], genetic markers, and glycolipid composition [20]. MAC strains display three different morphologies: smooth transparent, smooth opaque, and rough [18, 19], with the smooth variants being the most virulent morphology [18, 19]. In addition, MAC strains spread on solid hydrophilic surfaces through sliding motility mechanisms that are independent of extracellular structures [21, 22]. Bacterial motility plays a significant role in the colonisation of environmental surfaces and cells [21], which in turn has been correlated* in vitro* with the capacity to form biofilms on hydrophobic surfaces [23]. In* M. avium* strains, motility and biofilm formation have been correlated with colony morphology and the presence of glycopeptidolipids (GPLs) in the cell envelope [24, 25]. Specifically, smooth transparent* M. avium* variants show higher motility on hydrophilic surfaces and increased GPL production; conversely, rough variants show diminished motility and impaired GPL production [22].

GPLs are glycolipids attached to the outermost portion of some nontuberculous mycobacteria, including* M. avium*,* M. smegmatis*,* M. abscessus*, and* M. fortuitum* [25]. This type of glycolipid comprises a mixture of 3-hydroxy or 3-methoxy C_26_–C_34_ fatty acids amidated to a tripeptide-amino-alcohol (D-phenylalanine-D-*allo*-threonine-D-alanine-L-alaninol) [24, 25]. The lipopeptide core is subsequently glycosylated through 6-deoxytalose (linked to the* allo*-threonine) and *α*-L-rhamnose (linked to L-alaninol), and the resulting oligosaccharide residues are methylated to form the non-serovar-specific GPLs (nsGPLs) present in all GPL-producing mycobacteria. However, MAC strains also synthesise polar GPLs, in which some oligosaccharides residues are attached to the 6-deoxytalose producing serovar-specific GPLs (ssGPLs) [24, 25]. In* M. avium*, the consecutive actions of a peptide synthetase (PstA/PstB), a fatty acid synthase system (FAS) and polyketide synthetase (Pks), polyketide synthetase associated protein (PapA), glycosyl and methyl transferases (GtfA/GtfB/RtfA and MtfA/MtfB/MtfC/MtfD), and glycolipid transporters (TmtP and Gap) are responsible for the biosynthesis and subsequent translocation of GPLs to the cell wall surface [24–26].

Currently, the existence of GPLs in* M. colombiense* strains is completely unknown. In the present study, we showed that* M. colombiense* contains glycolipids with chromatographic behaviours similar to GPLs. In addition, this novel species forms biofilms on the hydrophobic surfaces of polystyrene, and motility is increased in strains displaying smooth colony morphology. Moreover, we examined the genes likely involved in GPL biosynthesis in the CECT 3035 strain.

## 2. Material and Methods

### 2.1. Bacterial Strains, Culture Conditions, and Genomic DNA Isolation

The* M. colombiense *clinical isolates 19B, 57B, the* M. colombiense* genome sequence strain CECT 3035,* M. avium* 104 [27], and* M. smegmatis* mc^2^155 [28] were used in this study (Table 1). Planktonic mycobacteria were cultured at 37°C with agitation (76 rpm) in Middlebrook 7H9 media supplemented with ADC (0.5% (w/v) bovine serum albumin, 0.2% (w/v) dextrose, 0.085% (w/v) NaCl, and 0.0003% (w/v) beef catalase) and 0.05% (v/v) glycerol, until an OD_600_ of 0.5 was obtained (planktonic conditions). For the cell motility assay, mycobacteria were cultured in motility medium containing 7H9 supplemented with ADC and 0.35% agarose.* Pseudomonas aeruginosa* ATCC27853 [29] cultured in motility medium was used as a positive control in the drop-collapsing test.

For DNA extraction, the mycobacteria were grown in 7H9-ADC broth to an OD_600_ of 0.5, centrifuged and resuspended in TE buffer (10 mM Tris-HCl and 1 mM EDTA, pH 8). Subsequently, the bacilli were inactivated through incubation at 80°C for 20 minutes. Genomic DNA was extracted using lysozyme, SDS/proteinase K, and CTAB/NaCl for cell disruption and chloroform : isoamyl alcohol (24 : 1, v/v) for getting rid of proteins [30, 31]. The DNA pellets were treated with DNAse-free RNAse resuspended in 0.1X TE, followed by quantification using a NanoDrop 2000c Spectrophotometer (Thermo Scientific, MA, USA). The DNA quality was assessed using agarose gel electrophoresis and spectrophotometry (OD_260_/OD_280_).

### 2.2. Cell Motility Assay and Congo Red Staining

For the cell motility assay, plates containing 7H9 media supplemented with ADC and 0.35% agarose were incubated at room temperature overnight prior to cell inoculation [21]. Subsequently, 3 *μ*L of mycobacterial culture at an OD_600_ of 0.6 (~2.7 × 10^5^ CFU) was carefully spotted onto the centre of the plates to minimise the spread of cells from the inoculation point. The plates were dried under sterile conditions for 30 min, sealed with Parafilm, and incubated at 37°C for 4-5 weeks under humidified conditions. The motility rate was recorded as the colony growth halo. The motility assays were performed in triplicate from three independent experiments using* M. avium* 104 as a control. Differences among the experimental data were compared using Student's* t*-test, and *P* < 0.05 was considered significantly different.

For the characterisation of colony morphology, plates containing 7H10-OADC (ADC + 0.05% oleic acid) media supplemented with 100 *μ*g/mL Congo Red (Sigma-Aldrich, MO, USA) [32] were inoculated as in the cell motility assay, and the resulting cultures were incubated for 4-5 weeks at 37°C under humidified conditions. The morphology was defined as rough or smooth, opaque or transparent, and yellow or beige.

### 2.3. Biofilm Formation Assay

Biofilm formation was assessed as previously described [22], with some modifications. Briefly, the wells of polystyrene microtiter plates (TPP, Switzerland) were filled with a mix of 75 *μ*L of planktonic mycobacterial culture (OD_600_ = 0.6) and 125 *μ*L of 7H9-ADC broth. The plates were incubated at 37°C for 6 weeks. Subsequently, the plates were washed three times with 1X PBS (0.14 M NaCl, 5 mM KCl, 10 mM Na_2_HPO_4_, and 2 mM KH_2_PO_4_), 200 *μ*L of 0.1% crystal violet solution was added, and the cells were incubated at 37°C for an additional 30 min. The residual crystal violet solution was removed, and the wells were washed three times with 1X PBS buffer. A total of 200 *μ*L of absolute ethanol was added to each well, and the plates were incubated for 1 hour at room temperature. Colouration was quantified at 570 nm using a iMark Microplate Absorbance Reader 168-1135 (BioRad, PA, USA). The biofilm assays were performed in triplicate from three independent experiments using* M. avium* 104 as a positive control. Differences among experimental data were compared using Student's* t*-test, and *P* < 0.05 was considered significantly different.

### 2.4. Drop-Collapsing Test

Mycobacteria grown on motility medium were scraped and resuspended in 5 mL 1X PBS, gently vortexed, and centrifuged at 8000 rpm for 1 h. Subsequently, 20 *μ*L of supernatant was carefully spotted onto the 15.4 mm wells of polystyrene plates (TPP, Switzerland) previously coated with 100 *μ*L of mineral oil and incubated for 24 h at room temperature [33, 34]. The aqueous drops were visually examined, incubated for 1 min at room temperature, and subsequently photographed. The tests were performed in triplicate from three independent experiments using deionised water and 1% SDS,* M. smegmatis* mc^2^155, and* P. aeruginosa* as controls.

### 2.5. Lipid Extraction and TLC Analysis

Crude mycobacterial lipid extracts were obtained as previously described [35], with some modifications. Briefly, mycobacteria cultured under planktonic and motility conditions were extracted using chloroform-methanol (1 : 2, v/v) through vigorous stirring for 48 h at room temperature, followed by chloroform-methanol (2 : 1, v/v) extraction under the same experimental conditions. Pooled and dried organic extracts were partitioned using a chloroform-methanol-water mixture (8 : 4 : 2, v/v/v). The organic phase was separated and evaporated to dryness, and the free glycolipids extracts were weighed. For GPL detection, 1 mg of the lipid extracts was dissolved in chloroform-methanol (2 : 1, v/v) at 20 mg/mL and analysed on 20 × 20 Silica Gel 60 TLC plates (Merck, NJ, USA) using chloroform-methanol-water (65 : 25 : 4, v/v/v) [35]. The carbohydrate-containing compounds were visualised after spraying the plates with 1% anthrone (Sigma-Aldrich, MO, USA) in sulphuric acid, followed by heating at 120°C. Crude* M. avium* 104 lipid extract was used as a control, and the glycolipid profile was obtained from three independent experiments performed in duplicate.

### 2.6. Bioinformatics Analysis

The genes implicated in the biosynthesis of GPLs in the* M. avium* 104 strain (http://www.ncbi.nlm.nih.gov/nuccore/CP000479.1) were used as model for the bioinformatics analysis. The gene sequences for* pstA*/*pstB* (peptide synthetases),* pks10* (polyketide synthetase),* gtfA*,* gtfB*, and* rtfA* (glycosyl transferases),* mtfA*,* mtfB*,* mtfC*, and* mtfD* (methyl transferases),* tmtpC*,* tmtpA*, and* tmtpB* (glycolipid transporters), and* papA2* (acyl transferase) (Table 1) were identified in* M. colombiense* CECT 3035 using the Genomic BLAST tool (http://www.ncbi.nlm.nih.gov/sutils/genom_table.cgi). The orthologous sequences identified in the* M. colombiense* CECT 3035 genome [36] were subsequently used as targets for primer design in the RT-qPCR experiments.

### 2.7. RNA Isolation and RT-qPCR Analysis

RNA of* M. colombiense* 19B, CECT 3035, and 57B cultured on both motility medium and planktonic conditions was isolated for RT-qPCR experiments. Mycobacteria grown on motility medium were scraped and resuspended in diethylpyrocarbonate- (DEPC-) treated water. Motile and planktonic mycobacteria were centrifuged, washed with DEPC-treated water, and resuspended in TRIzol (Invitrogen, USA). The cells were lysed using a Mini-Bead Beater 16 (BioSpec, OK, USA) and glass beads (0.1 mm). Total RNA was isolated as previously described [37], and quantified using a NanoDrop 2000c Spectrophotometer (Thermo Scientific, MA, USA). After DNase I treatment, the RNA quality was evaluated through agarose gel electrophoresis and spectrophotometry (OD_260_/OD_280_). cDNA libraries were constructed from 1 *μ*g of RNA using the RevertAid First cDNA Synthesis Kit (Fermentas, Lithuania). For reverse transcription, the samples were incubated at 37°C for 30 min, 42°C for 5 h, 72°C for 1 min, and 4°C for 10 min.

The primers used for the RT-qPCR analyses are listed in Table 1. The RT-qPCR reactions were set up in triplicate using the Express SYBR GreenER Universal qPCR SuperMix Kit (Invitrogen, NY, USA) and a CFX-96 thermocycler (Biorad, PA, USA) under conditions including a denaturation step for 5 min at 95°C, followed by 39 cycles of 95°C for 10 sec, 58°C for 10 sec, and 72°C for 15 sec. Primers or cDNA were omitted in the negative controls. The gene transcription was normalised to the mean value of 16S rRNA (*rrs*) gene expression. The transcription profile was obtained from three independent experiments performed in duplicate. For each experiment, the differences among experimental data were compared using Student's* t*-test, and *P* < 0.05 was considered significantly different.

## 3. Results

### 3.1. *M. colombiense *Is Motile and Forms Biofilms on Hydrophobic Polystyrene Surfaces

**Figure 1:**
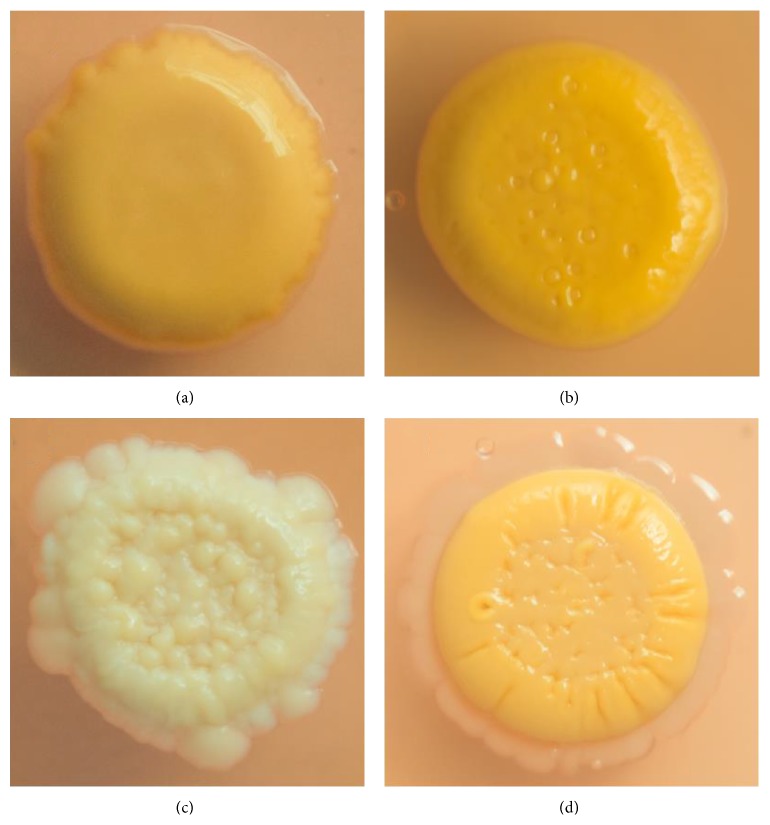
Colony morphology of* Mycobacterium colombiense* strains. Mycobacterial strains were grown on 7H10-OADC plates supplemented with Congo Red and incubated at 37°C for 4-5 weeks. (a)* M. colombiense* 19B, (b) CECT 3035, (c) 57B, and (d)* M. avium* 104.

The* M. colombiense* strains used in this study, including the genome sequence strain CECT 3035, presented smooth opaque and brilliant colonies on 7H10-OADC plates supplemented with Congo Red, except strain 57B, which displayed a rough and wrinkled phenotype (Figure 1). Similar results were obtained using medium lacking oleic acid (7H9-ADC-0.35% agarose, data not shown). Other* M. colombiense* strains not included in the present study (6B, 7B, 9B, and 16B) also displayed a smooth colony morphology (data not shown). Both smooth and rough bacterial phenotypes were stable after repetitive culture on solid media for the duration of the study (3 years). The smooth* M. colombiense* strains developed yellow pigmentation with age, specifically at the stationary phase, in all culture media; however, the rough variant (57B) remained beige in colour under the same experimental conditions (Figure 1).

**Figure 2:**
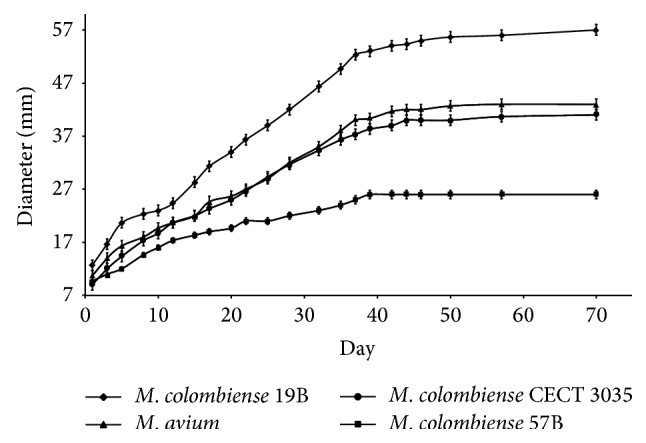
Motility rates for* Mycobacterium colombiense *strains. Mycobacterial strains were cultivated on 7H9-ADC-0.35% agarose and incubated at 37°C for 4-5 weeks. The colony growth halos were measured daily to determine the motility rate. Each reported value represents the mean of the colony growth halos from three independent experiments. The presented data have statistically significant differences compared with the values obtained from* M. avium *104 (*P* < 0.05).

Because alterations in the phenotypic characteristics associated with colony morphology have been observed in other MAC species, motility and biofilm formation were compared between smooth and rough* M. colombiense *strains. We observed that the* M. colombiense *19B and CECT3035 strains (smooth colony morphology) showed increased spreading (40.94 ± 0.05 *μ*m/day and 29.72 ± 0.05 *μ*m/day, resp.) on hydrophilic agarose compared with the natural rough variant 57B, which showed impaired spreading (Figure 2).

**Figure 3:**
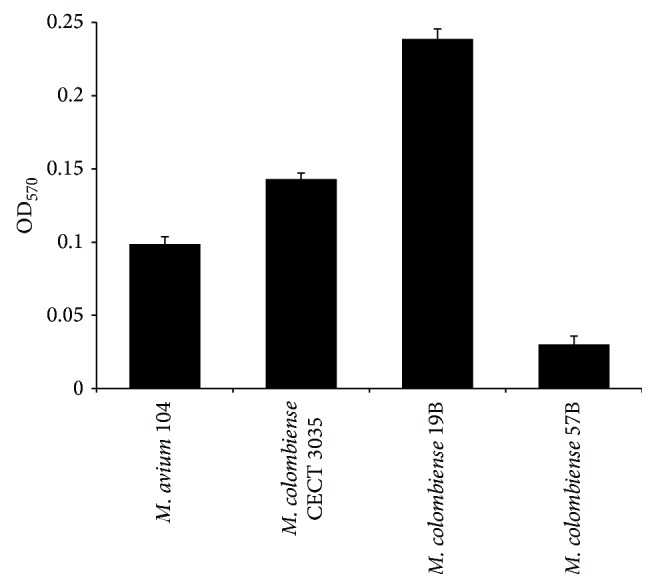
Capacity of* Mycobacterium colombiense *strains to form biofilms. The OD_570_ nm represents the proportion of mycobacteria added to the microtiter wells that reacted with the crystal violet solution. The bars represent the SD calculated from two independent experiments, each performed in triplicate.

Crystal violet staining was used to quantify the degree of* M. colombiense* biofilm formation on polystyrene. The highest OD_570_ values for all* M. colombiense* strains were obtained using an inoculum of ~2.7 × 10^5^ CFU/mL. In general, the strains displaying smooth morphology showed increased biofilm formation on hydrophobic surfaces, while the rough variant 57B showed reduced adhesion to polystyrene (Figure 3).

**Figure 4:**
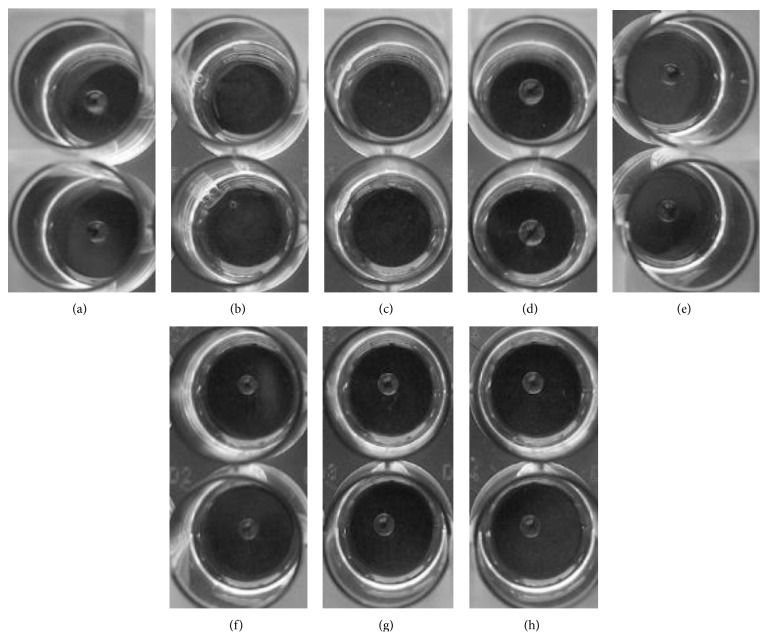
Drop-collapsing test. (a) Water (no surfactant), (b) 1% SDS (surfactant), (c)* P. aeruginosa*, (d)* M. smegmatis* mc^2^155, (e)* M. colombiense* 19B, (f)* M. colombiense* CECT 3035, (g)* M. colombiense* 57B, and (h)* M. avium *104. Drop spreading over the oily surface after samples incubation indicates the presence of biosurfactant substances in the aqueous sample. This assay was conducted in three independent experiments, each performed in duplicate.

As biosurfactants could potentially influence cell motility, the “drop-collapsing” method was used to detect biosurfactant secretion in* M. colombiense* strains [22]. As shown in Figure 4, drops of 1% SDS and* P. aeruginosa* aqueous extract (positive controls) spread over the oily surface after the samples were incubated, indicating the presence of biosurfactant substances. In contrast, drops of distilled water,* M. smegmatis* (negative control), and* M. colombiense* aqueous extracts did not collapse on the oily surface, but rather appeared as firm drops, suggesting the absence of biosurfactants in the culture supernatants.

### 3.2. *M. colombiense*,   Displaying a Smooth Colony Morphology, Contains Glycolipids with Thin-Layer Chromatographic Behaviour Similar to That of the GPLs of* M. avium*

**Figure 5:**
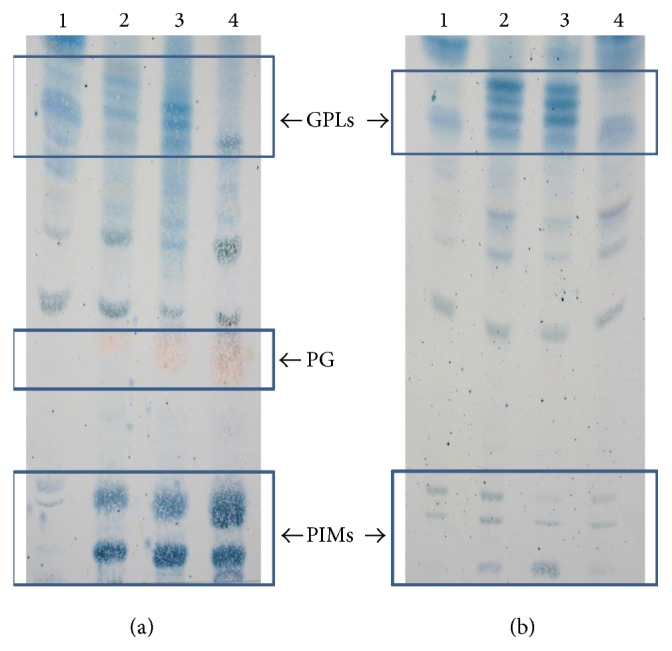
TLC profile of the GPLs in* Mycobacterium colombiense* strains. Crude lipid extracts of (1)* M. avium* 104, (2)* M. colombiense* 19B, (3)* M. colombiense* CECT 3035, and (4)* M. colombiense* 57B. Cells cultivated (a) under planktonic and (b) motile conditions were developed using chloroform-methanol-water (65 : 25 : 4, v/v/v). GPLs: glycopeptidolipids, PIMs: phosphatidyl-inositol mannosides, and PG: phosphatidylglycerol.

TLC analysis of noncovalently attached lipids extracted from mycobacteria cultured under planktonic and motile conditions showed that* M. colombiense* strains contain glycolipids with chromatographic behaviour similar to that of the GPLsof* M. avium* [23]. Regarding planktonic cells (Figure 5(a)), the strains with smooth colony morphology (19B and CECT 3035) showed multiple lipid spots migrating in the region of control GPLs (*M. avium* 104); however, the spots observed in the CECT 3035 lipid extract were more intense than those in the 19B strain. The natural rough variant (57B) showed only one spot that migrated in the same GPLs region.

Under motile conditions (Figure 5(b)), the smooth colony morphology strains 19B and CECT 3035 showed a GPL profile similar to that obtained for cells cultured under planktonic conditions; nevertheless, the counterpart spots were more intense than those observed in planktonic cells, particularly for the 19B strain. Nevertheless, the unique spot observed for motile 57B cells (rough variant) were more intense than that observed for planktonic 57B cells. Interestingly, all motile* M. colombiense* cells exhibited reduced production of polar glycolipids, such as phosphatidyl-inositol mannosides (PIMs) and phosphatidylglycerol (PG), compared with planktonic cells (Figure 5(b)).

### 3.3. *In Silico* Identification of GPL Biosynthesis Genes in* M. colombiense *CECT 3035

**Figure 6:**
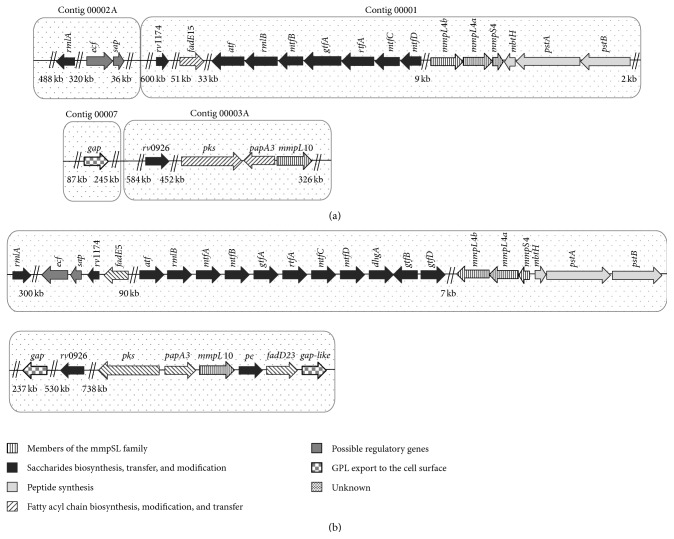
Model showing the gene cluster proposed for the GPL biosynthesis of* Mycobacterium colombiense* CECT 3035 (a). The location and identity of the* M. colombiense* genes were established using the previously described biosynthetic pathway for GPL biosynthesis annotated in the* M. avium* 104 genomic sequence (b).

The genes involved in GPL biosynthesis in* M. colombiense* are currently unknown. Based on the GPL biosynthetic pathway reported for* M. avium* 104 [25], 30 open reading frames (ORF), encoding peptide synthetase, fatty acid synthases (FAS), polyketide synthases (PKs), acyltransferase (PapA), glycosyltransferases, carbohydrate synthetases, methyl and acyl transferases, glycolipid transporters, potential biosynthesis regulators, and proteins with unknown functions [25, 26], were searched within the recently reported nucleotide sequence of* M. colombiense* CECT 3035 [36]. This* in silico* analysis (see Table S1 of the Supplementary Material available online at http://dx.doi.org/10.1155/2015/419549) showed that all enzymatic activities searched were encoded by different ORFs in* M. colombiense* CECT 3035 with an identity ranging between 77% and 96%. The ORFs in the genome of the* M. colombiense* counterpart were located in the contigs 00001, 00002A, 00003A, and 00007 of the genome sequence of the CECT 3035 strain (Figure 6). Among the 30 genes of the GPL synthetic pathway in* M. avium* 104, 7 genes, (*mtfA, gtfB, gtfD, dghA, fadD23, pe*, and* gap*-*like*) did not have orthologous sequences in* M. colombiense* CECT 3035 (Table S1).

### 3.4. Differential Transcription of the GPL Biosynthesis Genes in* M. colombiense *Displaying Smooth and Rough  Colony Morphologies

RNA was isolated from* M. colombiense* CECT 3035, 19B, and 57B strains cultured under planktonic and motile conditions to quantify the transcription levels of the* pstA, gtfA, rtfA, mtfB, mtfC, mtfD, tmtpC, tmtpA, *and* tmtpB* genes, predicted to encode key enzymes of the GPL biosynthetic pathway (Table S1). For relative quantification,* M. colombiense* CECT 3035 was grown to the exponential phase under planktonic conditions and used as the reference pattern for two reasons: (1) CECT3035 is the* M. colombiense* genome sequence strain [36] and, consistent with the TLC analysis, (2) planktonic cells displayed the lowest GPL production.

**Figure 7:**
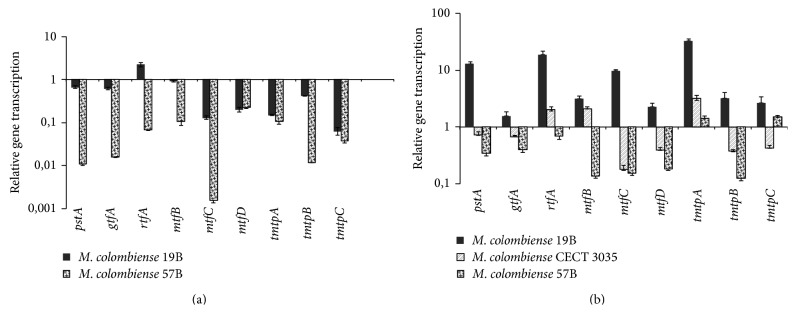
*In vitro* transcription of* Mycobacterium colombiense *genes involved in GPL biosynthesis. Relative quantification expressed as the ratio of gene transcription in (a)* M. colombiense *cells grown under planktonic conditions/transcription of* M. colombiense* CECT 3035 cells grown under planktonic conditions and (b)* M. colombiense* cells grown on the motility medium/transcription of* M. colombiense* CECT 3035 grown under planktonic conditions. The presented data have statistically significant differences compared with the values obtained from* M. colombiense* CECT 3035 cells (*P* < 0.05).

With regard to planktonic cells, the rate of* M. colombiense* 19B gene transcription was consistently higher than that for the* M. colombiense* 57B genes. Conversely, the rough variant (57B) displayed a dramatic decrease in the rate of transcription for the 9 selected genes, particularly those encoding peptide synthetase* pst*A, glycosyltransferase* gtf*A, the methyltransferases* mtf*B and* mtf*C, and the lipid transporters* tmtp*A,* tmtp*B, and* tmtp*C (Figure 7(a)). Interestingly,* rtfA* (rhamnosyltransferase) in the 19B strain (smooth colony morphology) was the only gene among the strains cultured under planktonic conditions that showed an increased transcription rate (2.21-fold higher) compared with the control cells (planktonic* M. colombiense* CECT 3035 at the exponential phase of growth).

Under motile growth conditions,* M. colombiense* 19B showed the highest transcription rate for all selected genes compared with the CECT 3035 and 57B strains (Figure 7(b)). In contrast, the transcription rates for all genes in the 57B strain (rough variant), except* tmtp*A and* tmtp*C (lipid transporters), were reduced. In motile CECT 3035 cells, whereas* rtfA, mtfB* (methyl transfers), and* tmtpA* showed increased transcription, the* pstA, gftA, mtfC, mtfD, tmtpB, *and* tmtpC* genes exhibited reduced transcription compared with control cells.

## 4. Discussion

In the present study, TLC experiments were able to show that* M. colombiense* contains glycolipids with chromatographic behaviour similar to the GPLs of* M. avium* 104, which have been previously identified and completely characterised [23]; in addition, the colony morphology was associated with the GPL profile of* M. colombiense* strains. Thus, the TLC analysis revealed that (1) strains displaying smooth colony morphology (19B and CECT3035) produce a higher amount of variable GPLs compared with the rough natural variant (57B) strain and (2) the GPL production was augmented in* M. colombiense* cells cultivated under the motile growth conditions. As expected, the TLC analyses did not provide detailed information about GPL structure; however structural determination was not the objective of the present study.

The media composition influences the glycolipid content of the mycobacterial cell wall. The MAC strains growing on solid medium exhibit a significant rate of transition from smooth transparent to smooth opaque and from smooth to rough morphologies [18, 19, 38]. We compared planktonic and motile* M. colombiense* cells grown in the same culture medium (7H9-ADC or 7H9-ADC-agarose) to avoid differences in lipid content produced through alterations in the media composition. The results showed that the low GPL content observed in the rough 57B variant is less frequent among* M. colombiense* strains; therefore,* M. colombiense* shows a preference for augmented GPL production and smooth colony morphology. Interestingly, planktonic* M. colombiense* cells displayed increased PIM and PG production and diminished GPL content compared with cells cultivated under motile conditions, suggesting a potential compensatory mechanism in cell wall lipid synthesis that compensates for the low GPL content.

The abundance of GPLs in the outermost portion of the cell envelope could be associated with the motility of* M. colombiense *strains on hydrophilic surfaces. Recht and others [22] proposed that GPLs are linked through the hydrophilic head to the cellular capsule and the hydrophobic fatty acid chain is exposed to the bacterial surface, thereby reducing interactions with hydrophilic agarose surfaces and facilitating the spread of cells on solid medium [21, 22]. Thus, the augmented GPL production of motile 19B and CECT 3035 cells compared with that of the rough variant 57B is consistent with the sliding motility on the hydrophilic agarose surface observed for these strains. It has also been suggested that bacterial sliding motility might be favoured through the secretion of surfactant substances from cells [21, 22]. The results of the drop-collapsing test showed that biosurfactants were likely not secreted from* M. colombiense* cells, suggesting that the motility of* M. colombiense* could be favoured through hydrophobic molecules, such as GPLs, bound to the outermost portion of cells; however, we cannot completely rule out the possibility that some of the surfactant substances secreted from* M. colombiense* remained adhered to the mycobacterial cell surface, thereby influencing cell motility.

GPLs on the outermost portions of the* M. colombiense* cell envelope generate a more hydrophobic cell surface that facilitates initial interactions with hydrophobic surfaces and increase biofilm formation on polystyrene wells. In addition, the increased GPL production in 19B and CECT 3035 cells is consistent with the augmented biofilm formation observed for* M. colombiense* strains displaying smooth colony morphology. Moreover, increased biofilm formation on hydrophobic surfaces and enhanced sliding over motility plates for 19B cells compared with the CECT 3035 strain, which also displays smooth colony morphology, likely suggest possible differences in GPL structure between* M. colombiense* strains. Differences in GPL production between strains could influence the* M. colombiense* ability to form biofilms on polyvinylchloride (PVC) pipes, which would facilitate the dissemination of these bacteria in natural environments, such as in-hospital spaces, thereby increasing the chance of infection in immunosuppressed patients [23, 39, 40].

Based on the GPL biosynthetic pathway previously described for the* M. avium* 104 strain [25], we are currently constructing a potential gene cluster for GPL biosynthesis in* M. colombiense*, which will be finished when the nucleotide sequence of the CECT 3035 is completely assembled. RT-qPCR experiments showed that the planktonic and motile cells of the rough 57B strain showed decreased transcription of the 9 selected genes, leading to the low production of GPLs in this rough variant compared with the* M. colombiense* strains displaying smooth colony morphology. Interestingly, planktonic 19B cells exhibited lower expression of most of the selected genes compared with planktonic CECT 3035 cells. This behaviour results in the reduced production of GPLs in planktonic 19B cells compared with the CECT 3035 strain, consistent with the results of the TLC experiments.

It has been previously shown that an* rtfA *mutation in the* M. avium* serovar-2-specific strain resulted in the loss of serovar-specific GPLs, thereby diminishing the variability of these glycolipids in the cell envelope [41, 42]. Thus, it is possible that the enzymatic activity of the RtfA protein could be relevant for the increased glycosylation and/or augmented production of ssGPLs in strains displaying smooth colony morphology compared with the rough variant 57B. In strains displaying smooth colony morphology, the genes encoding rhamnosyl and methyl transferases (*rtfA* and* mtfB*) are overtranscribed, suggesting the increased production of nsGPLs, precursors for ssGPL biosynthesis [24–26], thereby increasing the production of GPLs. The diminished transcription of* pstA, gftA, mtfC, mtfD, tmtpB*,and* tmtpC* in motile CECT 3035 compared with control cells (planktonic CECT 3035) is intriguing; however, this behaviour also suggests the relevance of rhamnosyl transferases, particularly RtfA, in GPL biosynthesis in* M. colombiense. *Nevertheless, the actual role for RtfA in* M. colombiense* strains should be confirmed using* rtfA-*null mutants.

We did not identify 7 of the 30 known GPL cluster genes of* M. avium* 104 in the genome sequence of* M. colombiense* CECT 3035. This interesting observation suggests that the GPLs between these two closely related MAC species could have different structural characteristics. However, further experiments using mass spectrometry and NMR are necessary to evaluate potential differences in the GPL structure among* M. colombiense* strains.

## 5. Conclusions

In conclusion, the results of the present study show that* M. colombiense* strains displaying smooth morphology exhibit increased biofilm formation on hydrophobic polystyrene surfaces and enhanced sliding over motility plates. Bioinformatics analyses indicate that the gene cluster established for GPL formation, modification, and translocation is intact, strongly suggesting that GPLs are putatively present in* M. colombiense*. In addition, motile culture conditions activate the transcription of the genes implicated in the key enzymatic activities of GPL biosynthesis.

## Supplementary Material

Supplementary Table: Alignment of the genes for the biosynthesis of Mycobacterium avium GPLs against the M. colombiense CECT 3035 nucleotide sequence.

## Figures and Tables

**Table 1:** Bacterial strains and primers used in this study.

Strain and primers	Description	Source
*M. colombiense* (CECT 3035)	Sequence genome strain	Clinical isolate
*M. colombiense* 19B and 57B	Clinical isolates	Clinical isolate
*M. smegmatis* mc^2^155	Reference strain	[28]
*M. avium* 104	Reference strain	[27]
*P. aeruginosa* ATCC27853	Reference strain	[29]

Primers	Sequence (5′-3′)	

*pstA* dir	ACAGGGCACGAGGAATTCTA	This study
*pstA* rev	TAGTCCTCGGAGGCTTCGTA	This study
*gftA* dir	ATGTGTGCTGGCCAGTTATG	This study
*gftA* rev	GGAAGAACGACGTCCAGAAG	This study
*rtfA* dir	GACTTTTGGAGCGACGAGTT	This study
*rtfA* rev	GCCAAATCCTGGTAAAGCTG	This study
*mtfB* dir	GGACACCGAGCACTACGAG	This study
*mtfB* rev	TCATACAGATCGCCATCCAG	This study
*mtfC* dir	ACAAGGCGGATAAAGGGATT	This study
*mtfC* rev	CTCATACAGATCGCCATCCA	This study
*mtfD* dir	TACCTGCTCGACACCTTCG	This study
*mtfD* rev	TCGACCTGCTCGAGTGTCT	This study
*tmtpC* dir	TTCATTCGGGATACCAGGAG	This study
*tmtpC* rev	TTGATCCTGACCCGAAGTTT	This study
*tmtpA* dir	CTCTCGGCTTTGACGACAC	This study
*tmtpA* rev	ATGGCCGACATCAGCTACTT	This study
*tmtpB* dir	GAGTGCCCTTGAGTGATTCC	This study
*tmtpB* rev	CCTCCAAGAATGACGATTCC	This study
16 sRNA dir	GAGATAGGCGTTCCCTTGTG	This study
16 sRNA rev	CTGGACATAAGGGGCATGAT	This study

## References

[1] Field S. K., Fisher D., Cowie R. L. (2004). *Mycobacterium avium* complex pulmonary disease in patient without HIV infection. *Chest*.

[2] Vaerewijck M. J. M., Huys G., Palomino J. C., Swings J., Portaels F. (2005). Mycobacteria in drinking water distribution systems: ecology and significance for human health. *FEMS Microbiology Reviews*.

[3] Hagiwara E., Komatsu S., Nishihira R., Shinohara T., Baba T., Ogura T. (2013). Clinical characteristics and prevalence of pneumothorax in patients with pulmonary *Mycobacterium avium* complex disease. *Journal of Infection and Chemotherapy*.

[4] Murcia M. I., Tortoli E., Menendez M. C., Palenque E., Garcia M. J. (2006). *Mycobacterium colombiense* sp. nov., a novel member of the *Mycobacterium avium* complex and description of MAC-X as a new ITS genetic variant. *International Journal of Systematic and Evolutionary Microbiology*.

[5] Tortoli E., Rindi L., Garcia M. J. (2004). Proposal to elevate the genetic variant MAC-A included in the *Mycobacterium avium* complex, to species rank as *Mycobacterium chimaera* sp. nov. *International Journal of Systematic and Evolutionary Microbiology*.

[6] Salah I. B., Cayrou C., Raoult D., Drancourt M. (2009). *Mycobacterium marseillense* sp. nov., *Mycobacterium timonense* sp. nov. and *Mycobacterium bouchedurhonense* sp. nov.,members of the *Mycobacterium avium* complex. *International Journal of Systematic and Evolutionary Microbiology*.

[7] van Ingen J., Boeree M. J., Kösters K. (2009). Proposal to elevate *Mycobacterium avium* complex ITS sequevar MAC-Q to *Mycobacterium vulneris* sp. nov. *International Journal of Systematic and Evolutionary Microbiology*.

[8] Bang D., Herlin T., Stegger M. (2008). *Mycobacterium arosiense* sp. nov., a slowly growing, scotochromogenic species causing osteomyelitis in an immunocompromised child. *International Journal of Systematic and Evolutionary Microbiology*.

[9] Cayrou C., Turenne C., Behr M. A., Drancourt M. (2010). Genotyping of *Mycobacterium avium* complex organisms using multispacer sequence typing. *Microbiology*.

[10] Smole S. C., McAleese F., Ngampasutadol J., von Reyn C. F., Arbeit R. D. (2002). Clinical and epidemiological correlates of genotypes within the *Mycobacterium avium* complex defined by restriction and sequence analysis of *hsp65*. *Journal of Clinical Microbiology*.

[11] Muwonge A., Kankya C., Johansen T. B. (2012). Non-tuberculous mycobacteria isolated from slaughter pigs in Mubende district, Uganda. *BMC Veterinary Research*.

[12] Falkinham J. O. (2009). Surrounded by mycobacteria: nontuberculous mycobacteria in the human environment. *Journal of Applied Microbiology*.

[13] Vuorenmaa K., Salah I. B., Barlogis V., Chambost H., Drancourt M. (2009). *Mycobacterium colombiense* and pseudotuberculous lymphadenopathy. *Emerging Infectious Diseases*.

[14] Esparcia Ó., Navarro F., Quer M., Coll P. (2008). Lymphadenopathy caused by *Mycobacterium colombiense*. *Journal of Clinical Microbiology*.

[15] Cohen-Bacrie S., David M., Stremler N., Dubus J.-C., Rolain J.-M., Drancourt M. (2011). *Mycobacterium chimaera* pulmonary infection complicating cystic fibrosis: a case report. *Journal of Medical Case Reports*.

[16] Poulin S., Corbeil C., Nguyen M. (2013). Fatal *Mycobacterium colombiense*/cytomegalovirus coinfection associated with acquired immunodeficiency due to autoantibodies against interferon gamma: a case report. *BMC Infectious Diseases*.

[17] Leguizamón J., Hernández J., Murcia M.-I., Soto C.-Y. (2013). Identification of potential biomarkers to distinguish *Mycobacterium colombiense* from other mycobacterial species. *Molecular and Cellular Probes*.

[18] Chatterjee D., Khoo K. H. (2001). The surface glycopeptidolipids of mycobacteria: structures and biological properties. *Cellular and Molecular Life Sciences*.

[19] Torrelles J. B., Ellis D., Osborne T. (2002). Characterization of virulence, colony morphotype and the glycopeptidolipid of *Mycobacterium avium* strain 104. *Tuberculosis*.

[20] Thorel M. F., David H. L. (1984). Specific surface antigens of SmT variants of *Mycobacterium avium*. *Infection and Immunity*.

[21] Martínez A., Torello S., Kolter R. (1999). Sliding motility in mycobacteria. *Journal of Bacteriology*.

[22] Recht J., Martínez A., Torello S., Kolter R. (2000). Genetic analysis of sliding motility in *Mycobacterium smegmatis*. *Journal of Bacteriology*.

[23] Carter G., Wu M., Drummond D. C., Bermudez L. E. (2003). Characterization of biofilm formation by clinical isolates of *Mycobacterium avium*. *Journal of Medical Microbiology*.

[24] Schorey J. S., Sweet L. (2008). The mycobacterial glycopeptidolipids: structure, function, and their role in pathogenesis. *Glycobiology*.

[25] Pang L., Tian X., Pan W., Xie J. (2013). Structure and function of mycobacterium glycopeptidolipids from comparative genomics perspective. *Journal of Cellular Biochemistry*.

[26] Mukherjee R., Chatterji D. (2012). Glycopeptidolipids: immuno-modulators in greasy mycobacterial cell envelope. *IUBMB Life*.

[27] Horan K. L., Freeman R., Weigel K. (2006). Isolation of the genome sequence strain *Mycobacterium avium* 104 from multiple patients over a 17-year period. *Journal of Clinical Microbiology*.

[28] Snapper S. B., Melton R. E., Mustafa S., Kieser T., Jacobs W. R. (1990). Isolation and characterization of efficient plasmid transformation mutants of *Mycobacterium smegmatis*. *Molecular Microbiology*.

[29] Radji M., Agustama R. A., Elya B., Tjampakasari C. R. (2013). Antimicrobial activity of green tea extract against isolates of methicillin-resistant *Staphylococcus aureus* and multi-drug resistant *Pseudomonas aeruginosa*. *Asian Pacific Journal of Tropical Biomedicine*.

[30] Helden P., Victor T., Warren R., Helden E. (2001). Isolation of DNA from *Mycobacterium tuberculosis*. *Mycobacterium tuberculosis Protocols*.

[31] van Kessel J. C., Marinelli L. J., Hatfull G. F. (2008). Recombineering mycobacteria and their phages. *Nature Reviews Microbiology*.

[32] Parrish N. M., Ko C. G., Dick J. D., Jones P. B., Ellingson J. L. E. (2004). Growth, Congo Red agar colony morphotypes and antibiotic susceptibility testing of *Mycobacterium avium* subspecies paratuberculosis.. *Clin Med Res*.

[33] Tugrul T., Cansunar E. (2005). Detecting surfactant-producing microorganisms by the drop-collapse test. *World Journal of Microbiology and Biotechnology*.

[34] Jain D. K., Collins-Thompson D. L., Lee H., Trevors J. T. (1991). A drop-collapsing test for screening surfactant-producing microorganisms. *Journal of Microbiological Methods*.

[35] Muñoz M., Lanéelle M.-A., Luquin M. (1997). Occurrence of an antigenic triacyl trehalose in clinical isolates and reference strains of *Mycobacterium tuberculosis*. *FEMS Microbiology Letters*.

[36] González-Pérez M., Murcia M. I., Landsman D., Jordan I. K., Marinõ-Ramírez L. (2011). Genome sequence of the *Mycobacterium colombiense* type strain, CECT 3035. *Journal of Bacteriology*.

[37] Betts J. C., Lukey P. T., Robb L. C., McAdam R. A., Duncan K. (2002). Evaluation of a nutrient starvation model of *Mycobacterium tuberculosis* persistence by gene and protein expression profiling. *Molecular Microbiology*.

[38] Barrow W. W., Brennan P. J. (1982). Isolation in high frequency of rough variants of *Mycobacterium intracellulare* lacking C-mycoside glycopeptidolipid antigens. *Journal of Bacteriology*.

[39] Costerton W., Veeh R., Shirtliff M., Pasmore M., Post C., Ehrlich G. (2003). The application of biofilm science to the study and control of chronic bacterial infections. *Journal of Clinical Investigation*.

[40] Recht J., Kolter R. (2001). Glycopeptidolipid acetylation affects sliding motility and biofilm formation in *Mycobacterium smegmatis*. *Journal of Bacteriology*.

[41] Eckstein T. M., Silbaq F. S., Chatterjee D., Kelly N. J., Brennan P. J., Belisle J. T. (1998). Identification and recombinant expression of a *Mycobacterium avium* rhamnosyltransferase gene (rtfA) involved in glycopeptidolipid biosynthesis. *Journal of Bacteriology*.

[42] Maslow J. N., Irani V. R., Lee S.-H., Eckstein T. M., Inamine J. M., Belisle J. T. (2003). Biosynthetic specificity of the rhamnosyltransferase gene of *Mycobacterium avium* serovar 2 as determined by allelic exchange mutagenesis. *Microbiology*.

