# Supplementary material for: Sliding Motility, Biofilm Formation, and Glycopeptidolipid Production in Mycobacterium colombiense Strains
Source: Biomed Res Int. 2015 May 28;2015:419549. doi: 10.1155/2015/419549 (PMC4477443; doi:10.1155/2015/419549)
Supplement: Supplementary file 1 — Supplementary Table: Alignment of the genes for the biosynthesis of Mycobacterium avium GPLs against the M. colombiense CECT 3035 nucleotide sequence. [file 419549.f1.docx]

**Table S1** Alignment of the genes implicated in the biosynthesis of *Mycobacterium avium* GPLs against the *M. colombiense* CECT 3035 nucleotide sequence. Thirty genes implicated in the biosynthetic pathway of *M. avium* 104 were searched in *the M. colombiense* CECT 3035 nucleotide sequence using the Genomic BLAST tool (<http://www.ncbi.nlm.nih.gov/sutils/genom_table.cgi>).

| ***M. avium* 104 gene /function/protein** | ***M. colombiense* CECT3035 Orthologue** | ***M. colombiense* CECT 3035 Localisation** | **Alignment score** | **Identity %** | **E-value** |
| --- | --- | --- | --- | --- | --- |
| ***mmps4*  (MAV_3247)**  Required for assembly of GPL synthesis enzymes in the cell membrane  **MmpS4 protein** | **MCOL_V218616**  membrane protein  **MmpS4 protein**  **ID:**  [EJO86937.1](http://www.ncbi.nlm.nih.gov/protein/400329438) | Contig 00001  (20977-21456) | 494 | 88 | 4e-^140^ |
| ***mmpL4a* (MAV_3248)**  Members of the MmpL family. Required for assembly of GPL biosynthases in the cell membrane  **Mmp14A protein** | **MCOL_V218621**  membrane protein  ID: [EJO86938.1](http://www.ncbi.nlm.nih.gov/protein/400329439) | Contig 00001  (21453-24344) | 2979 | 85 | 0.0 |
| ***mmpL4b* (MAV_3249)**  Members of the MmpL family. Required for assembly of GPL biosynthases in the cell membrane  Mmp14B protein | **MCOL_V218626**  mmpL4_2  membrane protein  ID: [EJO86939.1](http://www.ncbi.nlm.nih.gov/protein/400329440) | Contig 00001  (24491-27352) | 2983 | 86 | 0.0 |
| ***Rv1174*  (MAV_3362)**  conserved hypothetical protein  unknown function | **MCOL_V219166**  conserved hypothetical protein  unknown function  ID: [EJO87047.1](http://www.ncbi.nlm.nih.gov/protein/400329548) | Contig 00001  (146477-146815) | 510 | 94 | 3e^-145^ |
| ***rmlA*  (MAV_4820)**  glucose-1-phosphate thymidylyltransferase  **RmlA protein** | **MCOL_V201710**  glucose-1-phosphate thymidylyltransferase  ID: [EJO90905.1](http://www.ncbi.nlm.nih.gov/protein/400333411) | Contig 00002A  complement  (355904-356779) | 1192 | 91 | 0.0 |
| ***rmlB*  (MAV_3269)**  NAD dependent epimerase/dehydratase family protein  **RmlB Protein** | **MCOL_V218756**  NAD-dependent dehydratase  ID: [EJO86965.1](http://www.ncbi.nlm.nih.gov/protein/400329466) | Contig 00001  complement  (55306-56409) | 1020 | 84 | 0.0 |
| ***mtfA***  (**MAV_3268)**  3-O-methyltransferase  MtfA protein | Not found |  |  |  |  |
| ***mtfB* (MAV_3266)**  Rhamnose 4-O-methyltransferase  **MtfB protein** | **MCOL_V218751**  macrocin-O-methyltransferase  ID: [EJO86964.1](http://www.ncbi.nlm.nih.gov/protein/400329465) | Contig 00001  complement  (54258-55064) | 937 | 87 | 0.0 |
| ***mtfC*  (MAV_3261)**  Rhamnose 4-O-methyltransferase  **MtfC protein** | **MCOL_V218736**  macrocin-O-methyltransferase  ID: [EJO86961.1](http://www.ncbi.nlm.nih.gov/protein/400329462) | Contig 00001  complement  (50267-51070) | 931 | 88 | 0.0 |
| ***mtfD***  **(MAV_3260)**  Rhamnose 3-O-methyltransferase  **MtfD protein** | **MCOL_V218731**  **MtfD protein**  ID: [EJO86960.1](http://www.ncbi.nlm.nih.gov/protein/400329461) | Contig 00001  complement  49351-50106) | 887 | 88 | 0.0 |
| ***gtfA*  (MAV_3265)**  D-allo-threonine 6-deoxytalosyltransferase  **GtfA protein** | **MCOL_V218746**  glycosyl transferase family 1  ID: [EJO86963.1](http://www.ncbi.nlm.nih.gov/protein/400329464) | Contig 00001  complement  (52857-54125) | 1513 | 89 | 0.0 |
| ***gtfB*  (MAV_3258)**  L-alaninol rhamnosyltransferase  **GtfB protein** | Not found |  |  |  |  |
| ***gtfD* (MAV_3253)**  Glycosyl transferase  **GtfD protein** | Not found |  |  |  |  |
| ***rtfA*  (MAV_3262)**  putative glycosyl transferase | **MCOL_V218741**  Glycosyl transferases, related to UDP-glucuronosyltransferase  ID: [EJO86962.1](http://www.ncbi.nlm.nih.gov/protein/400329463) | Contig 00001  complement  (51165-52355) | 1208 | 85 | 0.0 |
| ***atf*  (MAV_3274)**  putative acyltransferase  **AtF protein** | **MCOL_V218771**  putative acyltransferase  ID: [EJO86968.1](http://www.ncbi.nlm.nih.gov/protein/400329469) | Contig 00001  complement  (59477-60640) | 808 | 81 | 0.0 |
| ***mbtH*  (MAV_3245)**  unknown function  MbtH-like protein | **MCOL_V218611**  MbtH-like protein  ID: [EJO86936.1](http://www.ncbi.nlm.nih.gov/protein/400329437) | Contig 00001  complement  (19953-20183) | 372 | 96 | 1e^-103^ |
| ***pstA*  (MAV_3244)**  non-ribosomal peptide synthase  **PstA protein** | **MCOL_V218606**  non-ribosomal peptide synthase  ID:EJO86935.1 | Contig 00001  complement  (9653-19858) | 11424 | 87 | 0.0 |
| ***pstB*  (MAV_3243)**  non-ribosomal peptide synthase  **PstB protein** | **MCOL_V218601**  non-ribosomal peptide synthase  ID:[EJO86934.1](http://www.ncbi.nlm.nih.gov/protein/400329435) | Contig 00001  complement  (2007-9656) | 9631 | 89 | 0.0 |
| ***gap*  (MAV_3059)**  Integral membrane protein. Required for GPL export  **Gap protein** | **MCOL_V222543**  hypothetical protein  ID: [EJO86736.1](http://www.ncbi.nlm.nih.gov/protein/400329236) | Contig 00007  (86965-87759) | 237 | 82 | 1e^-62^ |
| ***sap*  (MAV_4518)**  Sigma-associated protein  **Sap protein** | **MCOL_V200150**  DGPF domain-containing protein  ID: [EJO90593.1](http://www.ncbi.nlm.nih.gov/protein/400333099) | Contig 00002A  (35928-36626) | 545 | 86 | 2e^-155^ |
| ***ecf*  (MAV_4519)**  Sigma factor of the ECF family  **ECF protein** | **MCOL_V200155**  Predicted RNA polymerase sigma factor containing a TPR repeat domain  ID: [EJO90594.1](http://www.ncbi.nlm.nih.gov/protein/400333100) | Contig 00002A  (36630-37847) | 1266 | 86 | 0.0 |
| ***fadE5*  (MAV_3309)**  Fatty acid dehydrogenase  **FadE5 protein** | **MCOL_V218931**  Acyl-CoA dehydrogenases  **FadE15 protein**  ID: [EJO87000.1](http://www.ncbi.nlm.nih.gov/protein/400329501) | Contig 00001  (93583-95409) | 2362 | 90 | 0.0 |
| ***Rv0926***  **(MAV_2461)**  hypothetical protein  unknown function | **MCOL_V208010**  hypothetical protein  unknown function  ID: [EJO90119.1](http://www.ncbi.nlm.nih.gov/protein/400332624) | Contig 00003A  (927060-928142) | 1314 | 89 | 0.0 |
| ***pks*  (MAV_1763)**  polyketide synthases  **PkS protein** | **MCOL_V205565**  polyketide synthases  ID: [EJO89632.1](http://www.ncbi.nlm.nih.gov/protein/400332137) | Contig 00003A  (332140-343203) | 7116 | 79 | 0.0 |
| ***papA3*  (MAV_1762)**  condensation domain protein, acyltransferase  **PapA3 protein** | **MCOL_V205560**  condensation domain protein  ID: [EJO89631.1](http://www.ncbi.nlm.nih.gov/protein/400332136) | Contig 00003A  complement  (330370-331776) | 710 | 77 | 0.0 |
| ***mmpL10*  (MAV_1761)**  Transport protein  MmpL10 protein | **MCOL_V205550**  Transport protein  MmpL10  ID: [EJO89629.1](http://www.ncbi.nlm.nih.gov/protein/400332134) | Contig 00003A  (326135-329125) | 399 | 77 | 7e^-111^ |
| ***fadD23*  (MAV_1759)**  acyl-CoA synthase  **FadD23 protein** | Not found |  |  |  |  |
| ***pe*  (MAV_1760)**  hypothetical protein  unknown function | Not found |  |  |  |  |
| ***gap-*tipo (MAV_1758)**  conserved hypothetical protein: Integral membrane protein | Not found |  |  |  |  |
| ***dhgA* (MAV_3259)**  dehydrogenase DhgA | Not found |  |  |  |  |
